# Multiple Transcriptome Analyses Reveal the Selected lncRNA-mRNA and circRNA-mRNA Networks in HepG2 Cells Expressing Genotype 4 Swine Hepatitis E Virus ORF3

**DOI:** 10.3390/vetsci12121151

**Published:** 2025-12-02

**Authors:** Hanwei Jiao, Chi Meng, Lingjie Wang, Shengping Wu, Gengxu Zhou, Yubo Qi, Jianhua Guo, Yu Zhao, Zuoyong Zhou, Ling Gan, Jake Wen

**Affiliations:** 1 The College of Veterinary Medicine, Southwest University, Chongqing 402460, China; jiaohanwei@swu.edu.cn (H.J.); mengchi@email.swu.edu.cn (C.M.); guolicheng666@email.swu.edu.cn (L.W.); chemie@email.swu.edu.cn (S.W.); zgx973589243@email.swu.edu.cn (G.Z.); qyb314159@email.swu.edu.cn (Y.Q.); guo0619@swu.edu.cn (J.G.); zzyxnny@163.com (Z.Z.); 2 Institute of Animal Husbandry and Veterinary Medicine of Guizhou Academy of Agricultural Science, Ministry of Agriculture and Rural Affairs Key Laboratory of Crop Genitic Resources and Germplasm Innovation in Karst Region, Guiyang 550005, China; zhaoyu@gzsnky.wecom.work; 3 Center for Translational Cancer Research, Brown Foundation Institute of Molecular Medicine 1825 Pressler St., Suite 310, Houston, TX 77030, USA

**Keywords:** HEV, swine hepatitis E virus, circRNA-mRNA, lncRNA-mRNA

## Abstract

(1) Background: Swine hepatitis E (SHE) is a novel zoonotic disease caused by the swine hepatitis E virus. Open Reading Frame 3 (ORF3) is an important virulence protein of swine hepatitis E virus, which promotes the survival and replication of the virus within host cells by regulating the ERK pathway, modulating the transport of growth factors that affect apoptosis, and facilitating the transmission of death signals during HEV infection. (2) Methods: In our previous study, we used adenovirus-mediated overexpression of HEV-4 in swine ORF3 in HepG2 cells, extracted total RNA, and performed high-throughput lncRNA, circRNA, and transcriptome sequencing. Based on the role of ORF3 in regulating the innate immunity of host cells, maintaining ERK activity, inhibiting the transport of growth factors, and suppressing the immune response, the pathways and differentially expressed genes were screened. A clustering analysis was conducted on circRNAs and lncRNAs, which were significantly differentially expressed across the four pathways, and a regulatory network of circRNA-miRNA and lncRNA-mRNA was constructed. (3) Results: In this study, a total of 24 circRNAs and 4 lncRNAs were screened in HepG2 cells expressing ORF3 of HEV-4 in swine, including 8 circRNAs that regulate host cell innate immunity and 18 circRNAs that maintain ERK activity. Four circRNAs inhibited growth factor transport, and three circRNAs inhibited immune responses, predicting the regulatory networks of circRNA-miRNA and lncRNA-mRNA, respectively. (4) Conclusions: In this study, differential genes involved in regulating host cell innate immunity, maintaining ERK activity, inhibiting growth factor transport, and inhibiting immune response processes were successfully screened in HepG2 cells expressing ORF3 in HEV-4 in swine, and the regulatory networks of circRNA-miRNA and lncRNA-mRNA were predicted, respectively, which laid a foundation for further elucidating the function of swine hepatitis E virus ORF3 and elucidating the infection mechanism of swine hepatitis E virus.

## Simple Summary

Hepatitis E virus genotype 4 (HEV-4) is a novel zoonotic disease caused by the hepatitis E virus. Open reading frame 3 (ORF3) is an important virulence protein that can regulate the ERK pathway, control the transport of growth factors affecting cell apoptosis, and promote the survival and replication of the virus in host cells. In this study, in HepG2 cells expressing ORF3 of HEV-4, differentially expressed genes involved in regulating the host’s innate immunity, maintaining ERK activity, inhibiting growth factor transport, and suppressing immune responses were successfully screened. Additionally, circRNA-miRNA and lncRNA-mRNA regulatory networks were predicted, laying a foundation for further elucidating the function of HEV ORF3 and understanding the infection mechanism of the hepatitis E virus.

## 1. Introduction

Swine hepatitis E (HE) is a zoonotic infectious disease caused by the hepatitis E virus (HEV), belonging to the Hepeviridae family; the strains that cause zoonotic infections in humans are classified as Paslahepevirus balayani (Eukaryotic Virus A), with the genotypic markers HEV-1, HEV-2, HEV-3, and HEV-4. It is a single-stranded positive-sense RNA virus, and the virus particle measures from approximately 27 to 34 nm in length. Clinically, it is primarily characterized by jaundice and acute viral hepatitis, and it is a major cause of acute hepatitis in humans [1]. Nowadays, the hepatitis E virus has significantly impacted the normal lives of humans, while also threatening the sustainable development of livestock farming. Swine hepatitis E has become a public health problem that countries are paying close attention to. At present, the hepatitis E virus can be divided into eight genotypes, but the common ones are HEV-1, HEV-2, HEV-3, and HEV-4, among which HEV-3 and HEV-4 have an obvious zoonotic nature, which can be transmitted to humans after infecting domestic pigs, wild boars, and immunized animals [2,3].

The HEV viral genome contains three open reading frames, and ORF3, although the smallest open reading frame in the HEV genome, contains the recognition sequences of a variety of protein kinases, which are believed to play a key role in signaling and viral factor release [4,5,6]. The HEV ORF3 protein can promote cell survival and proliferation by regulating the ERK pathway, growth factor transport to regulate apoptosis, and the transmission of death signals in HEV infection. The ORF3 protein also inhibits the innate host response by weakening the response in the acute phase and increasing the secretion of immunosuppressive factors (such as a-1-microglobulin). It may also regulate cell energy homeostasis by affecting the iron ion metabolism and lipid metabolism of host cells, thereby facilitating the survival and replication of viruses in cells [3,7].

Circular RNAs (circRNAs) are a class of single-stranded, covalently closed non-coding RNAs that are synthesized primarily by a reverse splicing mechanism [8,9]. CircRNAs play a key role in maintaining cellular homeostasis by influencing gene transcription, translation, and post-translational processes, regulating the immune system, and interacting with mRNA, miRNAs, and proteins. The circRNAs act as miRNA sponges through a competitive endogenous RNA (ceRNA) network and are involved in host antiviral immune responses [10,11].

Non-coding RNAs play a key role in various biological processes, and can be divided into cis-regulated lncRNAs and trans-regulated lncRNAs according to the transcription sites and functional positions of lncRNAs. Cis-regulated lncRNAs can increase or inhibit the expression of target genes through a variety of mechanisms, and this function seems to be related to their enhancers. In addition, cis-regulated lncRNAs may be involved in the transcription of multiple target genes, and conversely, it seems that multiple lncRNAs may be involved in the consistent transcription of target genes [12]. The lncRNAs form gene regulatory networks by interacting with other biomolecules, such as transcription factors, miRNAs, messenger RNAs (mRNAs), and RNA-binding proteins. By regulating the transcription and translation of mRNAs, lncRNAs can participate in several important biological processes, including cell differentiation, cell proliferation, and cell protection programs. Therefore, the identification of lncRNA-mRNA regulatory networks will aid in revealing the functions and regulatory mechanisms of lncRNAs [13,14].

In this study, we utilized adenovirus-mediated overexpression of genotype 4 swine hepatitis E virus ORF3 in HepG2 cells to conduct circRNA, lncRNA, and transcriptome sequencing, followed by GO and KEGG functional enrichment analyses. We further identified four pathways regulated by ORF3 that are involved in modulating host cell innate immunity, maintaining ERK activity, inhibiting growth factor transport, and suppressing immune responses. This allowed us to establish circRNA-miRNA and lncRNA-mRNA regulatory networks, laying the foundation for further elucidation of the function of swine hepatitis E virus ORF3 and the mechanisms of swine hepatitis E virus infection.

## 2. Materials and Methods

### 2.1. Preparation of Recombinant Adenovirus ADV4-ORF3 and High-Throughput Sequencing of circRNA, lncRNA, and Transcriptome

HepG2 cells were purchased from the Shanghai Cell Bank, Chinese Academy of Sciences, and adenovirus ADV4-ORF3 and control ADV4-GFP were produced by GenePharma Co., Ltd. (Shanghai, China). HepG2 cells were infected after Sanger sequencing, and then total RNA samples were extracted for high-throughput sequencing of circRNA, lncRNA, and transcriptome. Adenovirus ADV4-ORF3-infected HepG2 cell samples were named AD-ORF3, and the control group comprised adenovirus ADV4-GFP-infected HepG2 cell samples, named AD-GFP. Biological replicates were set at the same time [15,16].

### 2.2. Bioinformatics Analysis

Based on the gene expression profiles of six samples (Ad_GFP1, Ad_GFP2, Ad_GFP3, Ad_ORF3_1, Ad_ORF3_2, and Ad_ORF3_3), the functional enrichment of differentially expressed genes was analyzed using Gene Ontology (GO) and Kyoto Encyclopedia of Genes and Genomes (KEGG). According to the function of ORF3 to regulate host cell innate immunity, maintain ERK activity, inhibit growth factor transport, and inhibit immune response, the pathways and differentially expressed genes (*p* < 0.05) were screened from GO and KEGG, respectively. Cluster analysis was performed on circRNAs and lncRNAs that were significantly differentially expressed in the four pathways, and the expression of circRNAs and lncRNAs in different samples was visualized through heat maps. The horizontal axis represents the sample, and the vertical axis represents circRNA, lncRNA, and mRNA. Different colors represent the expression of differentially expressed genes within the group, where red represents gene up-regulation, and blue represents gene down-regulation.

### 2.3. Prediction of Four Pathways: circRNA-miRNA and lncRNA-mRNA Regulatory Networks Influenced by HEV-4 in Swine ORF3 in HepG2 Cells

The cis-regulation of lncRNA target genes is mainly predicted according to the location relationship, and the differentially expressed lncRNAs and differentially expressed mRNAs in the upper and lower 100 kbp of the chromosome are defined. Based on the literature report, circRNA, as endogenous competitive RNA (ceRNA), affects the post-transcriptional regulatory function of miRNA, and this is used as a basis for in-depth analysis to provide a basis for subsequent experimental validation. lncRNAs and circRNAs were screened, and regulatory networks were constructed.

## 3. Results

### 3.1. GO and KEGG Function Enrichment Analysis of Four Pathways in circRNA and lncRNA Transcriptome Sequencing Files Were Performed

**Figure 1:**
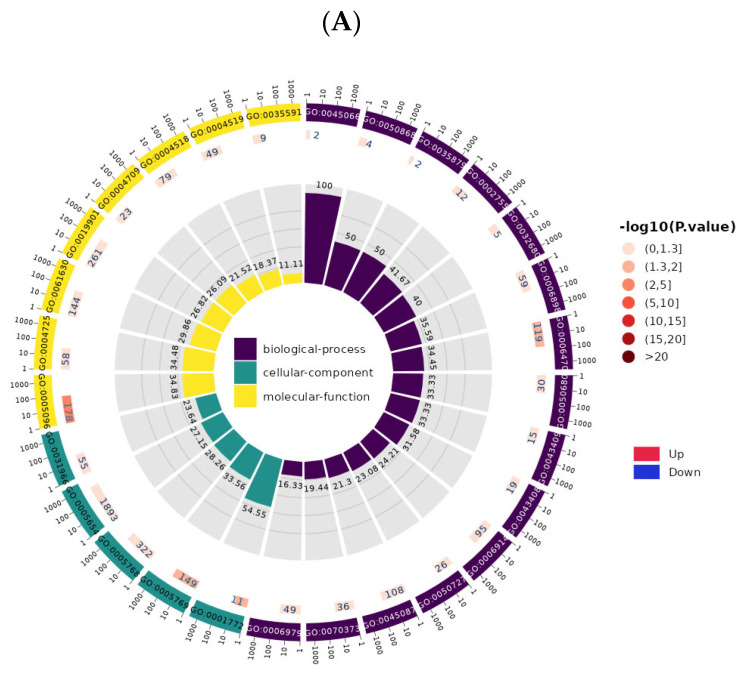

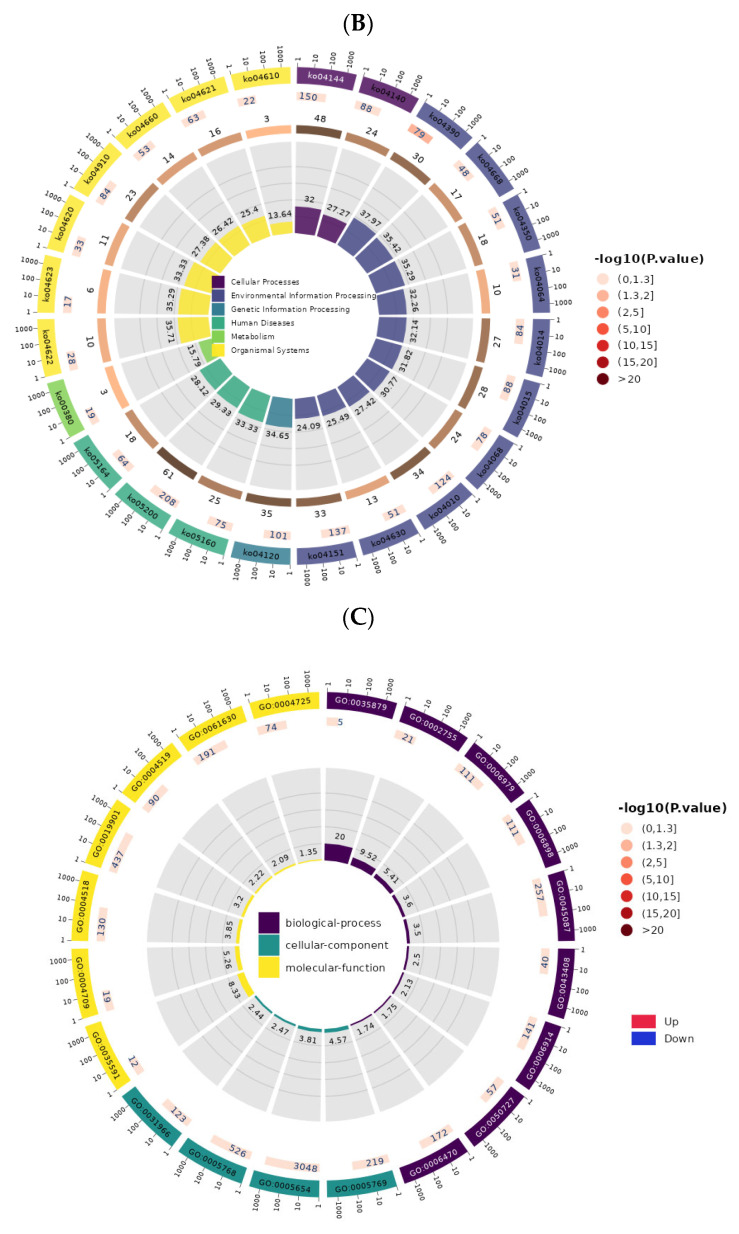

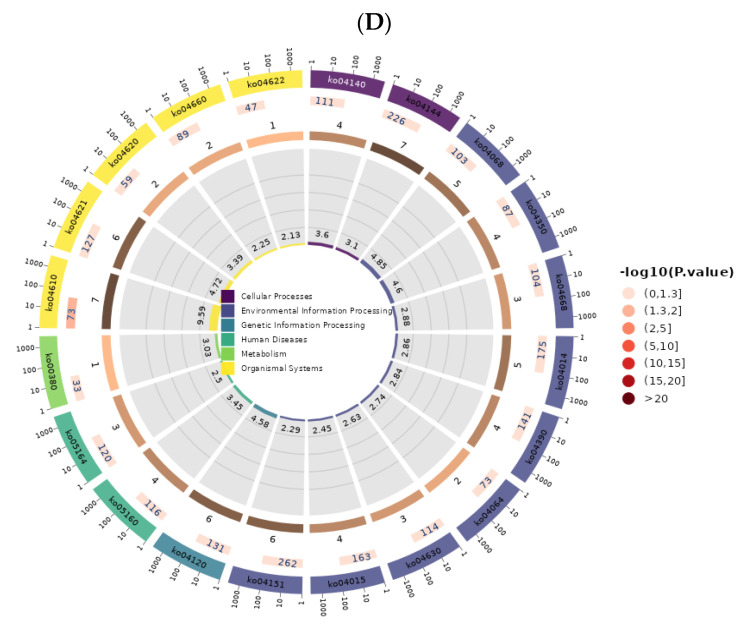
Enrichment circle plot of the four pathways exploited by viruses for immune evasion (regulating host cell innate immunity, maintaining ERK activity, inhibiting growth factor trafficking, and suppressing the immune response). (**A**): GO enrichment circos plot of circRNAs in the four pathways of viral immune escape. (**B**): KEGG enrichment circos plot of circRNAs in the four pathways of viral immune escape. (**C**): GO enrichment circos plot of lncRNAs in the four pathways of viral immune escape. (**D**): KEGG enrichment circos plot of lncRNAs in the four pathways of viral immune escape.

Gene Ontology (GO) and Kyoto Encyclopedia of Genes and Genomes (KEGG) were used to enrich and analyze the four functions involved in circRNA and lncRNA sequencing databases, including regulating host cell innate immunity, maintaining ERK activity, inhibiting growth factor transport, and inhibiting immune response (Figure 1, Table 1 and Table 2). According to the KEGG Level 1 summary of the selected pathways, it was found that the involved pathways are significantly enriched in Environmental Information Processing, all of which belong to signal transduction. These include the Jak-STAT signaling pathway (ko04630), NF-kappa B signaling pathway (ko04064), MAPK signaling pathway (ko04010), PI3K-Akt signaling pathway (ko04151), Ras signaling pathway (ko04014), Rap1 signaling pathway (ko04015), TNF signaling pathway (ko04668), TGF-beta signaling pathway (ko04350), FoxO signaling pathway (ko04068), and Hippo signaling pathway (ko04390). Following this are the pathways related to Organismal Systems, Human Diseases, Cellular Processes, Metabolism, and Genetic Information Processing. These pathways facilitate cell proliferation by maintaining ERK activity through the activation and inhibition of innate immunity while also preventing excessive release of growth factors and immune pathological damage, both of which hold key pathological significance in infections, cancers, and autoimmune diseases.

### 3.2. Differential Genes in circRNA and lncRNA Involved in Four Viral Immune Evasion Pathways

As previously described, in swine HEV-4 ORF3-mediated HepG2 cells, we revealed 62 significantly differentially expressed mRNAs (6564 transcripts), 319 lncRNAs (124 known lncRNAs and 195 novel lncRNAs), and 2261 circRNAs.

**Figure 2:**
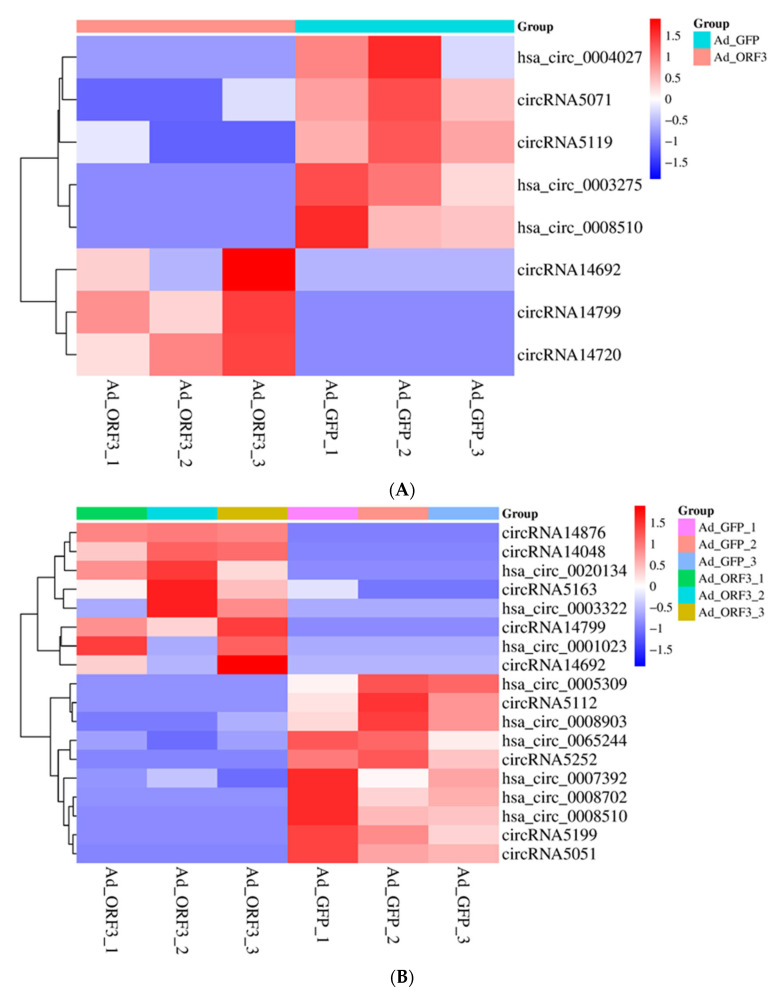

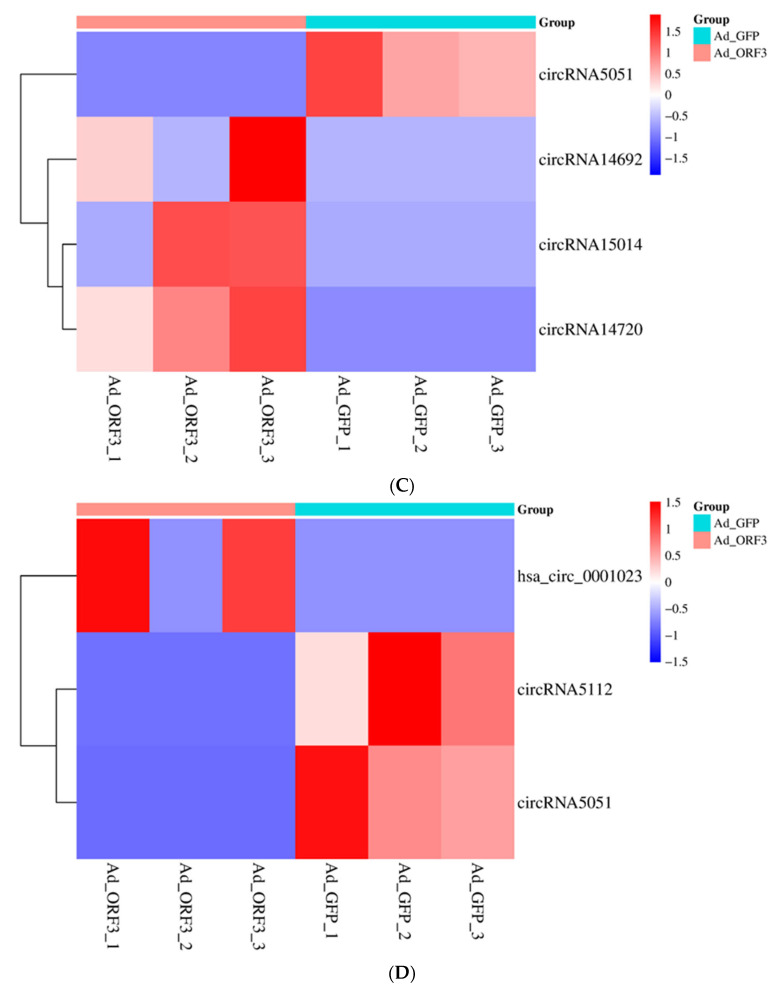

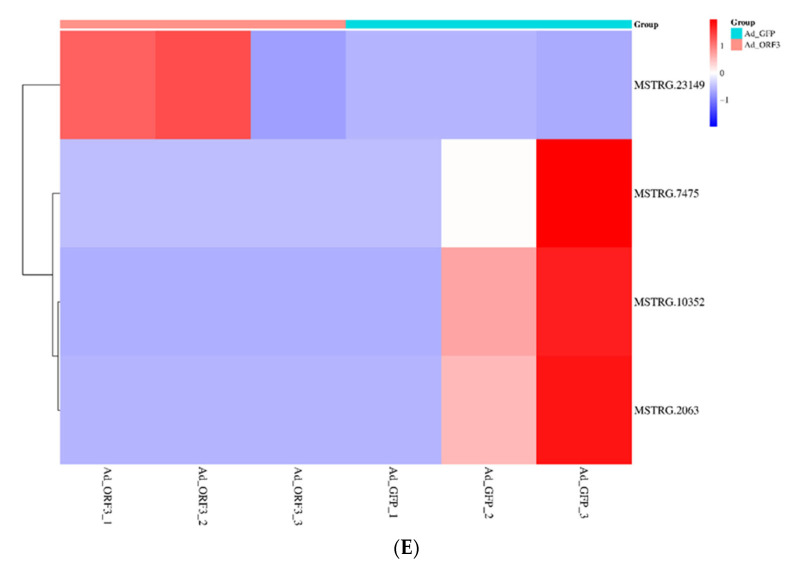
Differential genes in circRNA and lncRNA involved in four viral immune evasion pathways: regulating host innate immunity (**A**), maintaining ERK activity (**B**), inhibiting growth factor transport (**C**), and suppressing immune response (**D**) in circRNA; all four pathways in lncRNA (**E**).

In GO and KEGG, four pathways involved in regulating host cell innate immunity, maintaining ERK activity, inhibiting growth factor transport, and inhibiting immune response were screened, and pathway-related differential genes were obtained (*p* < 0.05). Among the circRNAs, a total of eight circRNAs were screened to participate in the innate immune pathway of host cells, including ENSG00000138279, ENSG00000108424, ENSG00000124198, ATP9B, VMP1, ENSG00000113273, ENSG00000196914, and UBQLN1. Among them, the expression of three genes was up-regulated, and five genes were down-regulated (Figure 2A).

A total of 18 circRNAs were screened to maintain the ERK active pathway, including GNB1, ENSG00000123983, ENSG00000175387, APLF, BRF1, TENT4A, ENSG00000129158, SMARCC1, ENSG00000108424, CACUL1, ENSG00000172530, ENSG00000169621, DCAF17, ENSG00000185658, ENSG00000196914, UBQLN1, PHF20, and DCAF5. Among them, the expression of eight genes was up-regulated, and ten genes were down-regulated (Figure 2B).

A total of four circRNAs were screened to inhibit the growth factor transport pathway, which were GPC3, ENSG00000196914, ENSG00000175387, and ENSG00000124198, respectively. Among them, the expression of three genes was up-regulated, and one gene was down-regulated (Figure 2C).

A total of three circRNAs were screened to inhibit the immune response pathway, which were APLF, ENSG00000169621, and ENSG00000175387. Among them, the expression of one gene was up-regulated, and the expression of two genes was down-regulated (Figure 2D).

In lncRNA, MSTRG.10352 plays a role in maintaining ERK activity and inhibiting growth factors, while MSTRG.2063 is capable of suppressing the transport of growth factors. MSTRG.23149 and MSTRG.7475 were identified in the screening of pathways that inhibit immune responses and regulate the innate immune pathways of host cells. Compared to the control group, the gene expression of MSTRG.23149 was up-regulated, while the expression of the other genes was down-regulated (Figure 2E).

### 3.3. Genotype 4 Swine Hepatitis E Virus ORF3 Rewires circRNA-miRNA and lncRNA-mRNA Networks in HepG2 Cells

In order to construct the circRNA-miRNA and lncRNA-mRNA networks, according to the description in Section 3.2, the network diagram of the four pathways related to the regulation of host cell innate immunity, the maintenance of ERK activity, the inhibition of growth factor transport, and the prediction of the target genes related to them were mapped.

**Figure 3:**
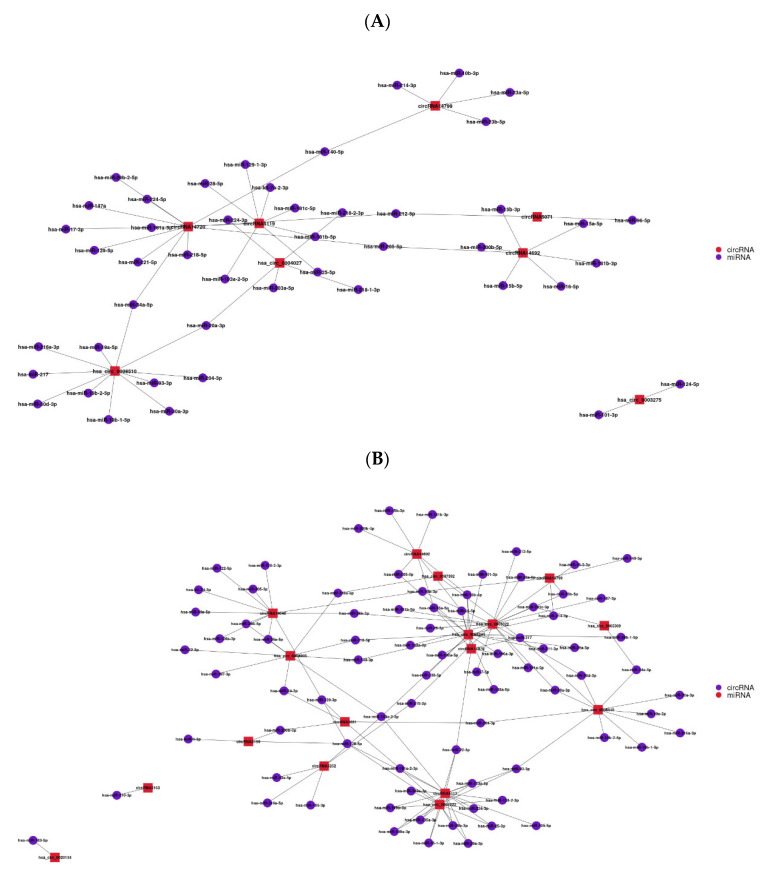

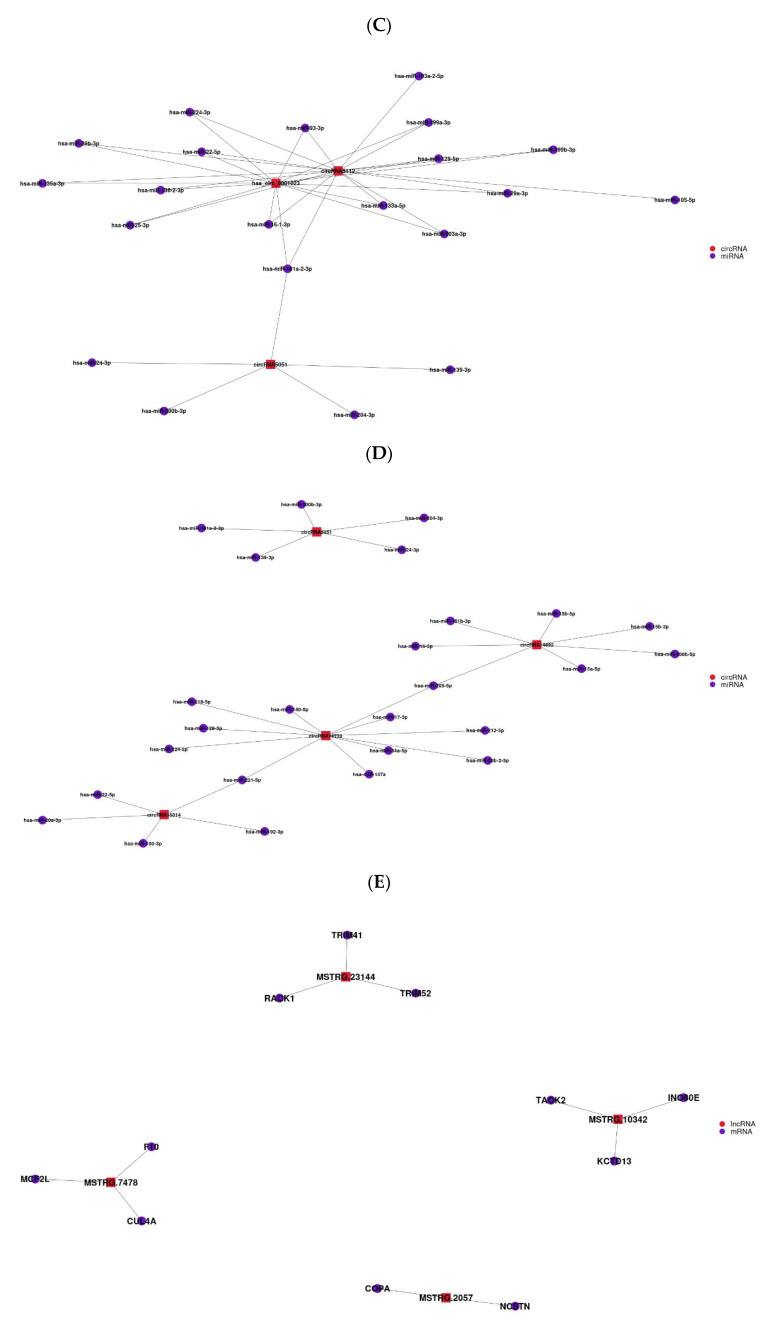
ircRNA-mRNA and lncRNA regulatory networks involved in host cell innate immune regulation, ERK activity maintenance, inhibition of growth factor transport, and inhibition of immune response processes. (**A**): A circRNA-miRNA regulatory network involved in the regulation of innate immune processes in host cells. (**B**): CircRNA-miRNA regulatory network involved in maintaining ERK activity. (**C**): CircRNA-miRNA regulatory network involved in inhibition of growth factor transport. (**D**): CircRNA-miRNA regulatory network involved in the process of inhibiting the immune response. (**E**): LncRNA-mRNA regulatory network involved in regulating host cell innate immunity, maintaining ERK activity, inhibiting growth factor transport, and inhibiting immune response processes.

The 8 circRNAs that regulated the innate immunity of host cells, 18 circRNAs that maintained ERK activity, 4 circRNAs that inhibited growth factor transport, and 3 circRNAs that inhibited immune response were predicted to predict their circRNA-miRNA regulatory networks, respectively (Figure 3).

The circRNA-miRNA regulatory network, which regulates innate immunity in host cells, contains a total of 8 circRNA nodes and 51 miRNA nodes (Figure 3A). The circRNA-miRNA regulatory network involved in maintaining ERK activity contains a total of 18 circRNA nodes and 132 miRNA nodes (Figure 3B). The circRNA-miRNA regulatory network that inhibits the transport of growth factors contains a total of 4 circRNA nodes and 28 miRNA nodes (Figure 3C). The circRNA-miRNA regulatory network that inhibits the immune response process contains 3 circRNA nodes and 37 miRNA nodes (Figure 3D).

Predicting the lncRNA-mRNA regulatory network for the four selected lncRNAs, KCTD13 (MSTRG.10342) may target and regulate TAOK2, INO80E, and KCTD13 itself. AL139011 (MSTRG.2057) may target and regulate COPA and NCSTN. AC138035 (MSTRG.23144) may regulate TRIM41, RACK1, and TRIM52. AL137002 (MSTRG.7478) may regulate F10, MCF2L, and CUL4A (Figure 3E).

## 4. Discussion

The hepatitis E virus (HEV) is the most common pathogen of acute hepatitis in the world, which can lead to chronic hepatitis or fulminant liver failure after infection, while pig infection with HEV causes swine hepatitis E, which causes zoonotic infectious diseases and seriously threatens the development of animal husbandry and public health. HEV ORF3 plays a key role in the longitudinal process of various host cells during viral infection and transmission, regulating multiple cell signaling pathways and inhibiting host immune responses to promote the survival of infected cells, creating an enabling environment for HEV replication and pathogenesis [17,18,19]. ORF3 plays a key role in overcoming cellular immunity in host cells and promoting HEV immune evasion. Studies have shown that ORF3 inhibits the secretion of THP1 macrophage inflammatory cytokines by inhibiting the activation of the NF-κB pathway, and can down-regulate TLR7 to impair the production of endogenous type I interferon in host cells, interfere with the host innate immune response, and maintain its own survival [7,20].

In this study, a total of 33 differential genes involved in the four pathways of regulating host cell innate immunity, maintaining ERK activity, inhibiting growth factor transport, and inhibiting immune response were screened in HepG2 cells expressing the genotype 4 porcine hepatitis virus ORF3, and a total of 24 differential circRNAs were screened from repeat data. Among them, ENSG00000196914, ENSG00000124198, ENSG00000108424, ENSG00000175387, ENSG00000169621, APLF, and UBQLN1 can participate in multiple pathways at the same time. The hosting genes of the miRNAs targeted by the most significant differential circRNAs in each pathway were screened to predict their potential functions, namely GNB1, GPC3, ENSG00000138279, and APLF, respectively. Research indicates that GNB1 can enhance cell proliferation, colony formation, cell migration, and invasion in vitro, and promotes the epithelial-to-mesenchymal transition process of HCC cells, thereby facilitating the progression of hepatocellular carcinoma through the activation of the P38/MAPK signaling pathway by targeting BAG2 [21]. GPC3 plays a significant role in anti-liver cancer cells. Sun et al. utilized GPC3-targeted CAR-T cells overexpressing GLUT1 or AGK for the treatment of HCC, which effectively and specifically lysed GPC3-positive tumor cells in an antigen-dependent manner in vitro. Furthermore, it was noted that this approach could activate the PI3K/Akt pathway to maintain the anti-apoptotic capabilities of CD8 T cells [22,23].

At the same time, four lncRNAs were screened, and an lncRNA-mRNA regulatory network was constructed. KCTD13 (MSTRG.10342) may target and regulate TAOK2 and INO80E. KCTD13 is involved in the MAPK signaling cascade and the inhibition of growth factor processes. Among the potentially targeted regulatory genes TAOK2, INO80E, and KCTD13, TAOK2 serves as a key member of the MAPK cascade, participating in the regulation of cell proliferation, apoptosis, migration, and invasion through various pathways. It also acts as a crucial regulator in Hippo signaling, playing a role in limiting tissue growth and proliferation [24,25]. In addition, INO80E and KCTD13 are also involved in MAPK signaling [26]. AL139011 (MSTRG.2057) may target the regulation of COPA and NCSTN. Research indicates that mutations in COPA are associated with autoimmune diseases and that COPA mutants induce the production and signaling of Type I IFN in a dominant-negative manner through STING, thereby controlling the autoimmune inflammation process [27]. NCSTN promotes the growth and metastasis of hepatocellular carcinoma cells by activating β-catenin in a Notch1/AKT-dependent manner [28]. AC138035 (MSTRG.23144) may regulate TRIM41, RACK1, and TRIM52, which are involved in immune responses. TRIM proteins can directly target viral components, regulate immune signaling pathways, and mediate ubiquitination modifications. TRIM41 activates the antiviral immune response by modifying BCL10 through lys63-linked polyubiquitination, centrally activating the NF-κB and TANK-binding kinase 1 (TBK1)-interferon regulatory factor 3 (IRF3) pathway. Studies indicate that RACK1 plays a crucial role in cancer progression, NF-κB activation, and various viral infections. Silencing RACK1 with siRNA suppresses PRRSV replication and persistent infection, eliminates PRRSV-induced NF-κB activation, and reduces viral titers [29]. AL137002 (MSTRG.7478) may regulate F10, MCF2L, and CUL4A. The expression level of the CUL4A gene is associated with viral replication. Research indicates that the knockdown of the CUL4 gene leads to the activation of the Jak-STAT pathway in mosquitoes, thereby reducing the replication of the virus in the body and saliva [30].

In this study, a total of 24 circRNAs and 4 lncRNAs were screened in HepG2 cells expressing ORF3 in genotype 4 porcine hepatitis virus, including 8 circRNAs that regulate host cell innate immunity and 18 circRNAs that maintain ERK activity. Four circRNAs inhibited growth factor transport, and three circRNAs inhibited immune responses, and predicted the regulatory networks of circRNA-miRNA and lncRNA-mRNA, respectively, which laid a foundation for further elucidating the function of swine hepatitis E virus ORF3 and elucidating the infection mechanism of swine hepatitis E virus. However, the functional predictions in this study are mainly based on bioinformatics analyses. Although GO and KEGG enrichment analyses provide strong clues about the potential functions of ORF3, such as its possible involvement in ERK signaling and immune regulation, these findings remain speculative. Future research will focus on experimentally validating these hypotheses, including the use of ORF3 mutants, pathway-specific inhibitors (such as MEK/ERK inhibitors), and techniques like Western blotting, to directly confirm the specific molecular mechanisms of ORF3.

## Figures and Tables

**Table 1:** GO of the four pathways by which viruses utilize immune evasion (regulating host cell innate immunity, maintaining ERK activity, inhibiting growth factor transport, and suppressing immune responses).

GO_ID	GO_Term
GO:0045087	Innate immune response
GO:0050727	Regulation of inflammatory response
GO:0006914	Autophagy
GO:0004518	Nuclease activity
GO:0005096	GTPase activator activity
GO:0005768	Endosome
GO:0031966	Mitochondrial membrane
GO:0035879	Plasma membrane lactate transport
GO:0002755	MyD88-dependent toll-like receptor signaling pathway
GO:0043409	Negative regulation of MAPK cascade
GO:0043408	Regulation of MAPK cascade
GO:0070373	Negative regulation of ERK1 and ERK2 cascade
GO:0006470	Protein dephosphorylation
GO:0006979	Response to oxidative stress
GO:0004725	Protein tyrosine phosphatase activity
GO:0019901	Protein kinase binding
GO:0004709	MAP kinase kinase kinase activity
GO:0005654	Nucleoplasm
GO:0050868	Negative regulation of T cell activation
GO:0045066	Regulatory T cell differentiation
GO:0032680	Regulation of tumor necrosis factor production
GO:0035591	Signaling adaptor activity
GO:0004519	Endonuclease activity
GO:0001772	Immunological synapse
GO:0006898	Receptor-mediated endocytosis
GO:0050680	Negative regulation of epithelial cell proliferation
GO:0061630	Ubiquitin protein ligase activity
GO:0005769	Early endosome

**Table 2:** KEGG of the four pathways by which viruses utilize immune evasion (regulating host cell innate immunity, maintaining ERK activity, inhibiting growth factor transport, and suppressing immune responses).

Pathway_id	Pathway_Name
ko04620	Toll-like receptor signaling pathway
ko04622	RIG-I-like receptor signaling pathway
ko04621	NOD-like receptor signaling pathway
ko04623	Cytosolic DNA-sensing pathway
ko04630	Jak-STAT signaling pathway
ko04064	NF-kappa B signaling pathway
ko04140	Autophagy-animal
ko04610	Complement and coagulation cascades
ko05164	Influenza A
ko05160	Hepatitis C
ko04010	MAPK signaling pathway
ko04151	PI3K-Akt signaling pathway
ko04014	Ras signaling pathway
ko04015	Rap1 signaling pathway
ko04910	Insulin signaling pathway
ko05200	Pathways in cancer
ko04668	TNF signaling pathway
ko04350	TGF-beta signaling pathway
ko04068	FoxO signaling pathway
ko04660	T cell receptor signaling pathway
ko00380	Tryptophan metabolism
ko04144	Endocytosis
ko04120	Ubiquitin mediated proteolysis
ko04390	Hippo signaling pathway

## Data Availability

The original contributions presented in this study are included in the article. Further inquiries can be directed to the corresponding author.
